# Efficacy of Flotetuzumab in Combination with Cytarabine in Patient-Derived Xenograft Models of Pediatric Acute Myeloid Leukemia

**DOI:** 10.3390/jcm11051333

**Published:** 2022-02-28

**Authors:** Sonali P. Barwe, Anne Kisielewski, Ezio Bonvini, John Muth, Jan Davidson-Moncada, Edward Anders Kolb, Anilkumar Gopalakrishnapillai

**Affiliations:** 1 Nemours Centers for Childhood Cancer Research, Nemours Children’s Hospital, Wilmington, DE 19803, USA; sbarwe@nemours.org (S.P.B.); akisielewski@hygeina.com (A.K.); eakolb@nemours.org (E.A.K.); 2 Nemours Center for Cancer and Blood Disorders, Nemours Children’s Hospital, Wilmington, DE 19803, USA; 3 MacroGenics, Inc., Rockville, MD 20850, USA; bonvinie@macrogenics.com (E.B.); muthj@macrogenics.com (J.M.); davidsonj@macrogenics.com (J.D.-M.)

**Keywords:** pediatric leukemia, acute myeloid leukemia, CD123, flotetuzumab, immunotherapy, patient-derived xenograft models

## Abstract

Children with acute myeloid leukemia (AML) have a poor prognosis despite the intensification of chemotherapy. Future efforts to improve outcomes should focus on more precise targeting of leukemia cells. CD123, or IL3RA, is expressed on the surface of nearly all pediatric AML samples and is a high-priority target for immunotherapy. The efficacy of an investigational dual-affinity retargeting antibody (DART) molecule (CD123 × CD3; MGD006 or flotetuzumab) was assessed in two distinct patient-derived xenograft (PDX) models of pediatric AML. MGD006 simultaneously binds to CD123 on target cells and CD3 on effector T cells, thereby activating T cells and redirecting them to induce cytotoxicity in target cells. The concurrent treatment of cytarabine and MGD006 was performed to determine the effect of cytarabine on T-cell counts and MGD006 activity. Treatment with MGD006 along with an allogeneic human T-cell infusion to act as effector cells induced durable responses in both PDX models, with CD123 positivity. This effect was sustained in mice treated with a combination of MGD006 and cytarabine in the presence of T cells. MGD006 enhanced T-cell proliferation and decreased the burden of AML blasts in the peripheral blood with or without cytarabine treatment. These data demonstrate the efficacy of MGD006 in prolonging survival in pediatric AML PDX models in the presence of effector T cells and show that the inclusion of cytarabine in the treatment regimen does not interfere with MGD006 activity.

## 1. Introduction

Children with acute myeloid leukemia (AML) suffer a poor prognosis, even though highly intensive chemotherapy is used for their treatment. Nearly a third of children with newly diagnosed AML and all children following disease relapse require myeloablative therapy with hematopoietic stem cell transplantation [1,2]. In order to improve outcomes, we must focus on the development and application of targeted therapies that can ultimately replace the significantly toxic standard-of-care.

Cell-surface CD123 (IL3RA) is expressed in nearly all AML samples, with increased expression in poor-prognosis tumors [3,4,5,6,7]. CD123 is a high-priority target for new therapy development in children [8,9]. There are active programs evaluating CD123-targeting antibody drug conjugates, bispecific antibodies, and chimeric antigen receptor T cells [10,11]. CD123 is expressed on the surfaces of AML blasts as well as leukemia stem cells [12], suggesting that targeting CD123 can eliminate leukemia stem-cell populations and achieve sustained remission [13]. A systematic analysis of CD123 expression levels in several pediatric AML patients revealed an association of higher CD123 expression with inferior clinical outcome and the presence of high-risk genetic aberrations, such as *KMT2A* rearrangements and FLT3-internal tandem duplications [4,5].

Flotetuzumab is an investigational CD123 dual-affinity retargeting antibody (DART) molecule (CD123 × CD3; MGD006) that simultaneously binds CD123 on target AML cells and CD3 on the effector T cells. This interaction activates T cells and redirects them to induce target-dependent cytotoxicity in AML blasts in vitro and in vivo via the secretion of granzyme and perforin [14,15]. Flotetuzumab showed promising clinical activity in adult patients with refractory AML [16]. A phase 1 trial of flotetuzumab in children is underway (NCT04158739), but preclinical data to further inform pediatric development are limited [17,18]. Herein, we evaluate MGD006 alone and in combination with cytarabine in patient-derived xenograft (PDX) models of pediatric AML. When administered to immune-deficient mice adoptively transferred with human allogeneic T cells, MGD006 improves mouse survival and induces T -cell proliferation when compared to treatment with either allogeneic T cells or MGD006 alone. Cytarabine does not attenuate allogeneic T-cell proliferation or the cytotoxic effect of MGD006.

## 2. Materials and Methods

### 2.1. Patient-Derived Xenograft Lines

The PDX lines NTPL-60, NTPL-146, NTPL-301, NTPL-377, NTPL-477, and NTPL-511 were generated and characterized as previously described [19,20]. PDX lines CBAM-44728-V1 and CBAM-68552-V1 (referred to as DF-5 and DF-2, respectively) were procured from Dana Farber Cancer Institute PRoXe depository [21]. All lines were generated from de-identified patient samples collected in accordance with a protocol approved by the Institutional Review Board. Patient characteristics and cytogenetics are included in Table S1.

### 2.2. Flow Cytometry and Quantitation of CD123 Cell Surface Expression

CD123 expression was detected using a PE-conjugated anti-human CD123 antibody (Clone 6H6, Catalog No. 306006, BioLegend; San Diego, CA, USA). Samples were analyzed on a NovoCyte 3000 Flow Cytometer. CD123 cell surface expression was quantitated with the use of BD Quantibrite PE Phycoreythrin Fluorescence Quantitation kit (Catalog No. 340495, BD Biosciences, San Jose, CA, USA) following the manufacturer’s protocol.

Peripheral blood was stained with APC-conjugated anti-mouse CD45 (BioLegend Catalog No. 103112), Pacific blue conjugated anti-human CD45 (BioLegend Catalog No. 304029) and FITC conjugated anti-human CD3 (BioLegend Catalog No. 317306) antibodies following incubation with human BD Fc block (BD Biosciences, Catalog No. 564219) to prevent non-specific antibody binding.

### 2.3. Leukemia Xenograft Models

NTPL-511 required injection of 2.5 × 10^6^ cells into the tail vein of NSG-SGM3 mice, while NTPL-146 engraftment required 2.0 × 10^6^ cells in NSG-B2m mice. No pre-conditioning treatment was performed before cell injection. After 18 days and 26 days post-transplant for NTPL-511 and NTPL-146, respectively, CD45+ human cells were detectable in mouse blood. Allogeneic human donor T cells (3 × 10^6^ cells per mouse) from StemCell Technologies (Cat No. 70024.1) were used to assess cell-mediated cytotoxicity in these immunodeficient mouse strains. T cells were injected via the intravenous route 3–4 h prior to MGD006 or vehicle control.

Following the injection of PDX cell lines and upon detection of human CD45+ cells, mice were randomly assigned to one of seven treatment groups: (1) untreated, (2) MDG006 (0.5 mg/Kg, Q5d), (3) cytarabine (50 mg/Kg, Q5d), (4) T cells, (5) T cells with MGD006 (0.5 mg/Kg, Q5d), (6) T cells with cytarabine (50 mg/Kg, Q5d), and (7) T cells with concurrent administration of cytarabine (50 mg/Kg, Q5d) and MGD006 (0.5 mg/Kg, Q5d). The MGD006 dose was established following previously published data [15]. Mice were monitored daily and peripheral blood was collected at least weekly to evaluate leukemia progression (CD45+CD3−) and T cell expansion (CD3+CD45+) by flow cytometry. Mice were euthanized when experimental endpoints consistent with systemic signs of leukemia based on weight and body condition score were reached or at the end of the experiment on day 250. Mice were considered to have durable response if the percentage of human CD45+ in peripheral blood was consistently lower than 3%, as described previously [22].

### 2.4. Statistical Analysis

Primary outcome measures include the rise in human leukemia (CD45+CD3−) cell percentage in mouse blood and survival estimates according to Kaplan–Meier estimator. GraphPad Prism 7 was used for statistical analysis. The differences in leukemia burden and T cell proliferation were evaluated for statistical significance using a two-tailed Student’s *t*-test (two-samples, with unequal variance). Overall survival was compared with the Log-rank (Mantel–Cox) test.

## 3. Results

### 3.1. CD123 Expression in Pediatric AML PDX Lines

**Figure 1:**
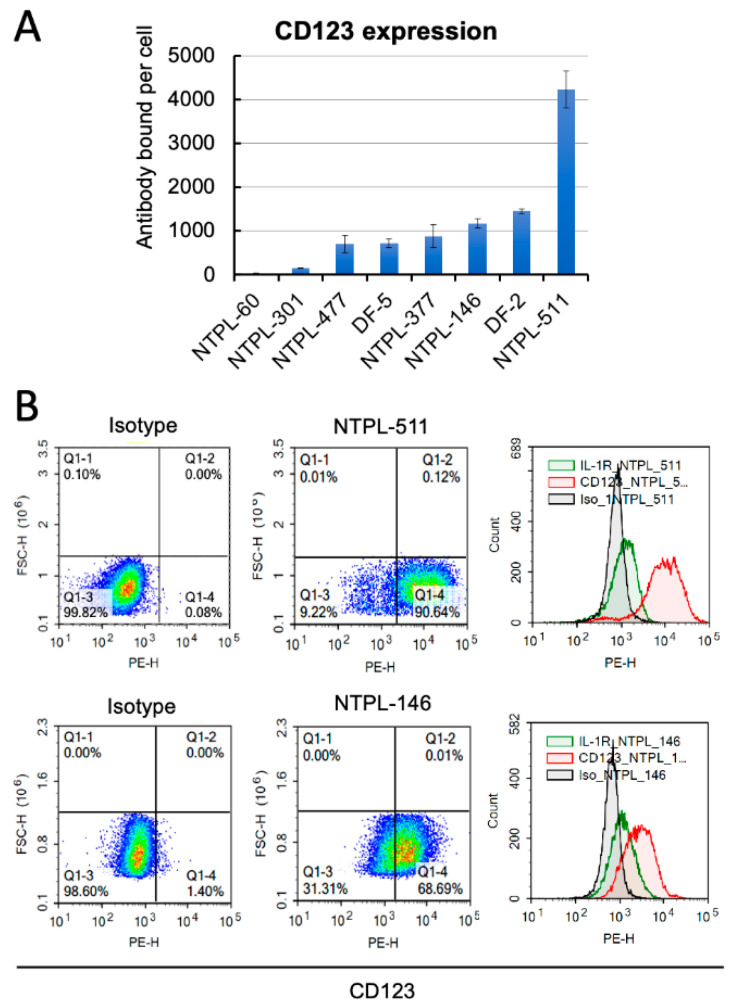
CD123 expression in pediatric AML PDX lines. (**A**) Graph shows the estimation of the number of antibody molecules bound per cell using Quantibrite fluorescence quantitation kit (BD Biosciences). Error bars denote SD of the mean from two independent experiments. (**B**) Flow cytometry plots showing PDX lines stained with PE-conjugated CD123 antibody or isotype control antibody.

CD123 expression on the cell surface was evaluated in a panel of pediatric AML PDX lines generated in the laboratory (Table S1). Consistent with previous reports [4], we observed that CD123 was expressed on the cell surface of the majority of pediatric AML samples according to a monomodal distribution (Figure 1A). Based on the Quantibrite methodology [23], NTPL-511 with the highest, and NTPL-146 with an intermediate level of surface CD123 (Figure 1B) were selected for the preclinical evaluation of MGD006, a CD123 × CD3 DART molecule.

### 3.2. Early AML Blast Reduction and T-Cell Expansion following MGD006 Treatment in Pediatric AML PDX Models

**Figure 2:**
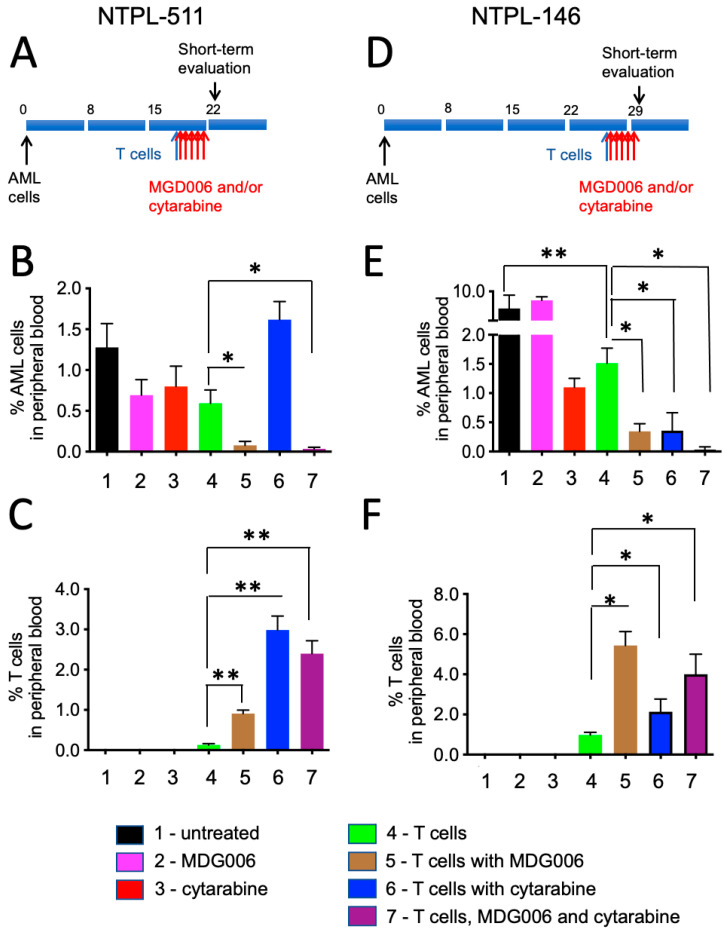
Short-term effect of MGD006 treatment in pediatric AML PDX models. (**A**,**D**) Schematic showing timeline of treatment. (**B**,**C**) Peripheral blood was collected on day 22 (treatment ended day 21) from NTPL-511 mice to determine AML (CD45+ CD3−) and T-cell (CD45+ CD3+) percentage by flow cytometry. (**E**,**F**) Peripheral blood collected from NTPL-146-transplanted mice on day 31 (treatment completed day 30) was used for calculating percentage of AML and T cells by flow cytometry. * *p* < 0.05, ** *p* < 0.01. The X-axis in B–F indicates treatment group numbers (1) untreated, (2) MDG006, (3) cytarabine, (4) T cells, (5) T cells with MGD006, (6) T cells with cytarabine, and (7) T cells with concurrent administration of cytarabine and MGD006.

The effect of MGD006 either alone or in combination with cytarabine in the presence of effector T cells was evaluated in PDX models (Figure 2A,D). Cohorts of mice receiving T cells, cytarabine, MGD006, T cells with cytarabine, or no treatment served as experimental controls. Figure 2B provides the results for the NTPL-511 PDX model. The mice injected with MGD006 only (group 2), cytarabine only (group 3), or T cells only (group 4), did not show a significant reduction in NTPL-511 cell load compared to the untreated mice (group 1) on day 22, when treatment ended (Figure 2B). The AML cell percentage in peripheral blood was significantly reduced in the mice receiving T cells + MGD006 (group 5, *p* = 0.046) or T cells + cytarabine + MGD006 (group 7, *p* = 0.041) compared to T cells only (group 4). Figure 2C demonstrates that exposure to MGD006 alone (group 5, *p* = 0.001) or in combination with cytarabine (group 7, *p* = 0.005) heightened the expansion of adoptively transferred T cells comparative to AML PDX mice receiving T cells only (group 4). T cell expansion without a reduction in AML cell percentage was observed when allogeneic T cells were injected with cytarabine but without MGD006 (group 6, Figure 2B,C).

In the NTPL-146 model (Figure 2E), the AML cell load in the peripheral blood was greatly reduced in all the treatment groups on day 30 (following treatment cessation) compared to untreated mice (group 1), except MGD006 (group 2). There was a significant drop in AML cell load in the mice treated with T cells + MGD006 (group 5, *p* = 0.014), T cells + cytarabine (group 6, *p* = 0.015), and T cells + cytarabine + MGD006 (group 7, *p* = 0.010) compared to the mice receiving T cells alone (group 4). Similar to the NTPL-511 model, the T-cell percentage was higher in the mice receiving T cells with MGD006 (group 5, *p* = 0.021), cytarabine (group 6, *p* = 0.073), or cytarabine + MDG006 (group 7, *p* = 0.029) compared to T cells alone (group 4) (Figure 2F). The reduction in AML blasts percentage mirrored the expansion of the T cells.

Taken together, these data from an early time point showed that MGD006-enhanced T cell expansion with or without cytarabine significantly reduced the AML blast percentage in peripheral blood.

### 3.3. Long-Term Leukemia Control and Survival following Treatment with MGD006 in Pediatric AML PDX Models

**Figure 3:**
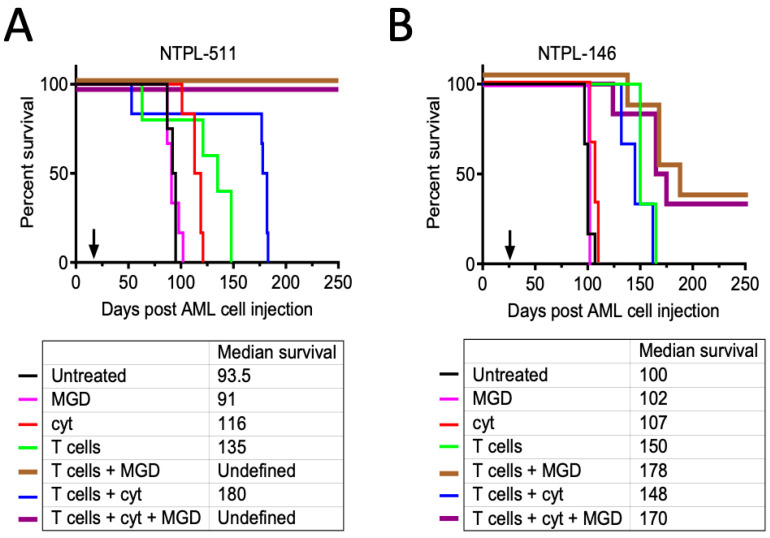
Efficacy of MGD006 in pediatric AML PDX models. (**A**,**B**) Kaplan–Meier survival plots showing the median survival. Arrow indicates time when treatment began.

MGD006 alone (group 2) had a minimal effect on reducing leukemic burden or survival compared to the untreated mice (group 1) in the PDX line with high CD123 expression (Figure 3A and Figure S1A) and intermediate expression (Figure 3B and Figure S1B), likely because of the need for the presence of effector human T cells. T-cell injection (group 4) delayed AML growth and improved median survival by 41.5 and 50 days in NTPL-511 (*p* = 0.030) and NTPL-146 (*p* = 0.009), respectively, compared to group 1, possibly due to the allogeneic effect of the T cells. MGD006 in the presence of effector T cells (group 5) reduced leukemic burden and prolonged survival compared to T cells alone (group 4). In the NTPL-511 model, the group 5 mice showed sustained low AML cell load (Figure S1A), and these mice did not meet the experimental endpoints at study termination after 250 days (Figure 3A, *p* = 0.001 in comparison with group 4). Survival in NTPL-146 was improved modestly (38-day increased median survival over group 4, *p* = 0.045) accompanied by delayed leukemia progression (Figure 3B and Figure S1B). Cytarabine treatment (group 3) slowed leukemia progression with a 22.5-day improvement in median survival comparative to the untreated mice in NTPL-511 (Figure 3A, *p* = 0.001, Figure S1A), while it had no significant effect in NTPL-146 (Figure 3B, *p* = 0.073, Figure S1B). Consistent with cytarabine-induced early T-cell expansion (Figure 2F), median survival in the mice treated with T cells + cytarabine (group 6) increased by 86.5 days compared to group 4 (*p* = 0.021). This effect was not observed in NTPL-146, as there were no significant differences in median survival between groups 4 and 6. The NTPL-511 mice receiving MGD006 concurrently with cytarabine following T-cell injection showed a durable response (Figure 3A, group 7), similar to the mice belonging to group 5. These group 7 mice had significantly lower amounts of residual AML cells in their peripheral blood compared to group 5 when the study was terminated (Figure S1A, 0.1% and 1.6% AML cells, respectively, *p* = 0.008). The group 7 mice also had significantly reduced bone marrow engraftment at study termination compared to the group 5 mice (Figure S1E, *p* = 0.0323). Two of six NTPL-146 mice belonging to groups 5 and 7 showed a durable response until 250 days (Figure 3B), indicating that cytarabine treatment did not hamper the anti-tumor activity and T-cell expansion mediated by MGD006. In the NTPL-146 mice, the AML cell percentage at the end of the study was lower in group 7 compared to group 5 (Figure S1B, 0.5% and 2.4% AML cells respectively (*p* = 0.013). Our data demonstrate MGD006’s activity in the presence of effector T cells in inducing a durable response in pediatric AML PDX models. Although the addition of cytarabine to this regimen did not alter median survival, it showed a statistically significant reduction in AML burden.

### 3.4. T-Cell Kinetics in Pediatric AML PDX Models

In addition to evaluating the AML cell load in the peripheral blood, we also checked the T-cell percentage. Both PDX models showed a rapid rise in T-cell counts, peaking approximately 30 days post-T-cell injection (Supplementary Figure S3C,D). T cells were detected in the peripheral blood of the NTPL-511 and NTPL-146 mice several days after treatment completion and throughout the course of the experiment.

## 4. Discussion

We evaluated the efficacy of flotetuzumab (MGD006) either as a single agent or administered concurrently with cytarabine in two pediatric AML PDX models with high or moderate CD123 expression. Adoptively transferred allogeneic T cells served as effector cells and a T-cells-alone group was included as a control to account for any allogeneic effect. Our data show the efficacy of flotetuzumab in pediatric AML PDX models. The concurrent administration of cytarabine did not attenuate T-cell expansion or inhibit MGD006 activity.

Our data show a durable response of MGD006 in all six of the mice engrafted with NTPL-511, a PDX model with high CD123 expression. In a PDX model with intermediate CD123 expression, NTPL-146, two of the six mice treated with MGD006 + T cells survived until study termination at 250 days. Although it is tempting to speculate that there is an association between CD123 expression and efficacy, systematic studies with large numbers of pediatric samples are needed to validate this correlation. However, unlike CD33- and CD38-targeting immunotherapies [24,25], no correlation between CD123 target expression and response was observed in a recent study with 88 adult AML patients [16]. It remains to be determined whether the CD123 expression in the leukemia stem cell population rather than bulk leukemia is indicative of response.

CD123 expression is detected on the surface of normal hematopoietic stem and progenitor cells, albeit at low levels compared to AML blasts [13]. Although the ablation of normal hematopoiesis was observed in one preclinical study using CD123-targeted chimeric antigen receptor T cells [26], other reports showed only a minimal effect [27,28]. Furthermore, preclinical and clinical data from CD123-targeting antibody-based approaches, including flotetuzumab, support no evidence of myelosuppression [15,16,29], indicating that MDG006 is likely to be safe for use in pediatric AML.

The mechanism of MGD006-mediated improvement in median survival and reduction in AML burden was based on combined DART-molecule- and T cell-mediated AML cell death, because MGD006 alone or T cells alone were ineffective or minimally effective, respectively, the latter due to an allogeneic effect. We acknowledge that this is a potential limitation of the study, as we are unable to rule out that the allogeneic effect may be necessary for the response in this model. MGD006, however, can mediate anti-leukemia activity in vitro via the engagement of autologous T cells in adult AML patient samples [14,15], suggesting that a prerequisite is not necessary for an allogeneic response. T cells were detectable in the peripheral blood at the time of euthanasia (up to 250 days), suggesting that a single injection of T cells is sufficient and durable in these PDX models.

Chemotherapy may enhance the anti-tumor effect of the immune system by acting on immune cell differentiation and antigen presentation [30], and by altering the immunosuppressive microenvironment in AML by reducing the regulatory T cell population [31]. Cytarabine, a component of the standard-of-care chemotherapy regimen, was used in our study in combination with MGD006. Our data indicate that cytarabine enhanced T cell counts, consistent with previous reports showing the stimulation of T-cell expansion and T-cell-mediated killing by cytarabine [17,32]. Although the inclusion of cytarabine did not alter median survival, the terminal percentage of AML cells was lower in the T cells + cytarabine + MGD006 group compared to the T cells + MGD006 mice. Taken together, our results provide the preclinical rationale for the clinical evaluation of MGD006 in pediatric AML with CD123 expression.

## Data Availability

Data presented in this article is available in here and in the Supplementary Materials.

## Supplementary Materials

The following supporting information can be downloaded at: https://www.mdpi.com/article/10.3390/jcm11051333/s1, Figure S1: Efficacy of MGD006 in pediatric AML PDX models. (**A**,**B**) Growth curve showing the rise in AML cell percentage in peripheral blood in NTPL-511 and NTPL-146 transplanted mice. (**C**,**D**) Time course of T cell percent population in peripheral blood in NTPL-511 and NTPL-146 engrafted mice. (**E**) Bone marrow engraftment in indicated mice at the time of euthanasia. * *p* < 0.05; Table S1: Patient characteristics, cytogenetics, and gene fusions of pediatric AML PDX lines.
