# Supplementary material for: Efficacy of Flotetuzumab in Combination with Cytarabine in Patient-Derived Xenograft Models of Pediatric Acute Myeloid Leukemia
Source: J Clin Med. 2022 Feb 28;11(5):1333. doi: 10.3390/jcm11051333 (PMC8911345; doi:10.3390/jcm11051333)
Supplement: Supplementary file 1 [file jcm-11-01333-s001.zip › jcm-1584084-supplementary.pdf]

Table S1: Patient characteristics, cytogenetics, and gene fusions of pediatric AML PDX lines

Xenograft	Age (years) / Sex	Sample collected at	Karyotype	FISH	Fusion
DF-2	1/M	Relapse	46,XY,inv(6)(q23q27)[20]	*KMT2A* rearrangement at 11q23	KMT2A-MLLT4
DF-5	15/F	Relapse	46,XX,inv(10)(p12.2q21.2)[20]	*KMT2A* rearrangement at 11q23 (88.0% of cells)	KMT2A-MLLT10
NTPL-60	4/M	Diagnosis	46, XY der (14;21) (q10;q10) ?c, +21c [cp12]/ 48, idem, +8, +der (14;21) (q10; q10) [cp8} - MDS related clone detected	apparent trisomy 8 and trisomy/tetrasomy 21; nuc ish 8q22 (ETOx3), 21q22 (AML1x3) [28/100] 8q22 (ETOx3), 21q22 (AML1x4) [57/100]	
NTPL-146	10/F	Diagnosis	46, XX, t(11;19)(q23;p13;3)[13]/46,idem,i(8)(q10)[7]	*KMT2A* rearrangement (97% cells) with evidence of clonal evolution	KMT2A-MLLT1
NTPL-301	13/F	Secondary	42~43, X, t(2;16)(q21;p13.1),add(4)(q21),der(5)t(5;12)(q13;q11.2),-7,add(12)(p11.2),add(15)(q22,-17,-19,add(20)(p13),+1~2mar[cp8/42,sl,-13[cp5]/42,sdl1,+del(13)(q12q14),-add(20)[2]/42,sdl2,-der(5),+add(7)(q22), ins(10)9p11.2)[2]/42, sl2,-7,der(13)t(7;13)(q11.2;p11.2)[2]/45,X,-X[1]	Monosomy5 (11%)/deletion of 5q (77.5%), Monosomy7 (51.5%)/deletion of 7q (31.5%), Loss of TEL/12p DNA sequence (76.5%)	

NTPL-377	1.5/F	Diagnosis	46, XX, t(9;11)(p21;q23)[20]	*KMT2A* rearrangement (87% cells)	KMT2A-MLLT3
NTPL-477	19/M	Relapse	46, XY[23] / del(9)(q13q22) and del(12)(p11.2p13)[3]	Not available	KMT2A-FLNB
NTPL-511	14/M	Diagnosis	47, XY,+8[1]/46,XY[29]	No assay specific abnormalities detected by AML FISH Panel. Trisomy 8 in 3% of cells.	NUP98-NSD1

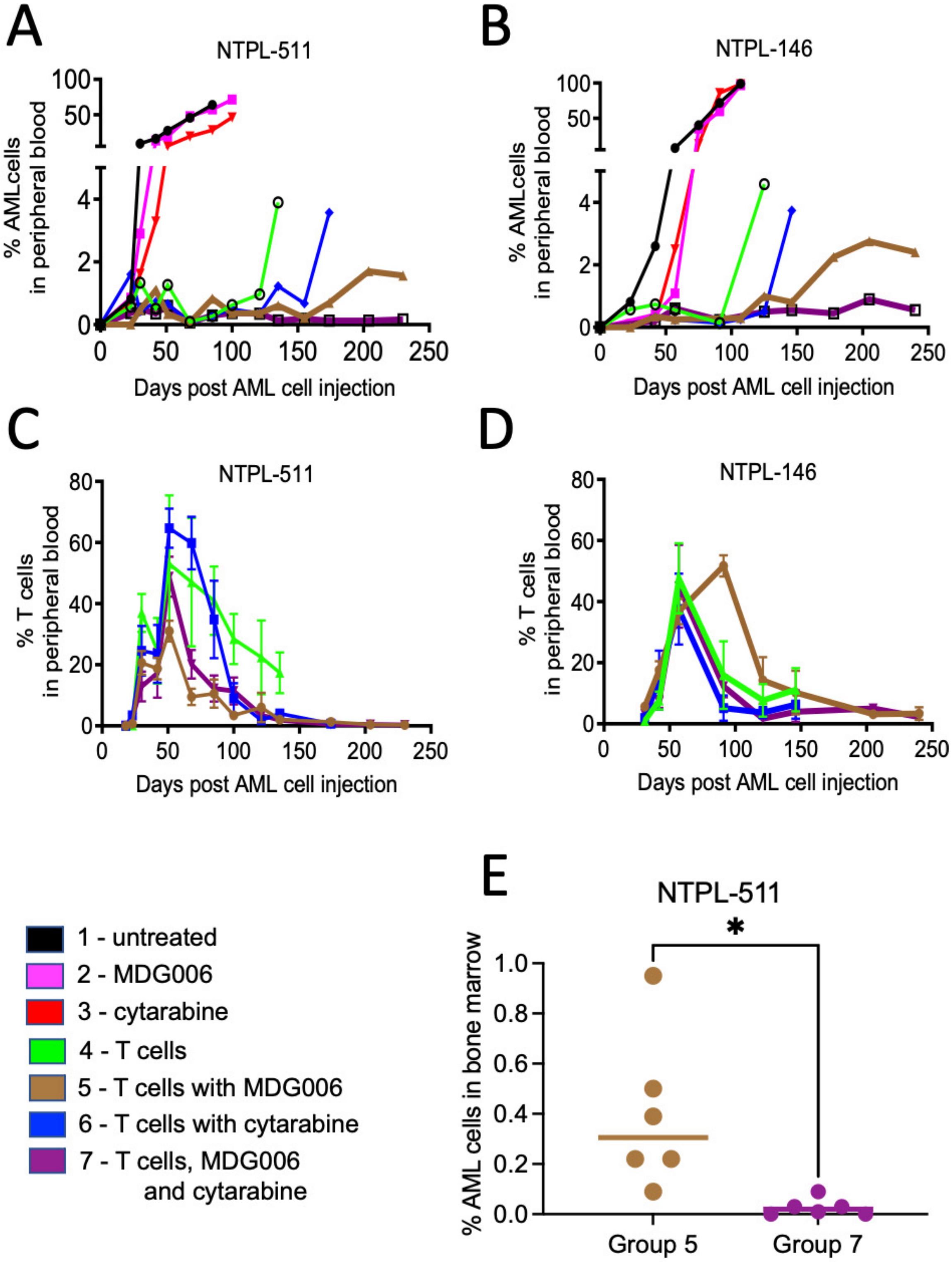
A
NTPL-511
% AMLcells in peripheral blood
Days post AML cell injection
B
NTPL-146
% AMLcells in peripheral blood
Days post AML cell injection
C
NTPL-511
% T cells in peripheral blood
Days post AML cell injection
D
NTPL-146
% T cells in peripheral blood
Days post AML cell injection
1 - untreated
2 - MDG006
3 - cytarabine
4 - T cells
5 - T cells with MDG006
6 - T cells with cytarabine
7 - T cells, MDG006 and cytarabine
E
NTPL-511
*
% AML cells in bone marrow
Group 5
Group 7

Figure S1. Efficacy of MGD006 in pediatric AML PDX models. **(A,B)** Growth curve showing the rise in AML cell percentage in peripheral blood in NTPL-511 and NTPL-146 transplanted mice. (**C,D)** Time course of T cell percent population in peripheral blood in NTPL-511 and NTPL-146 engrafted mice. (**E)** Bone marrow engraftment in indicated mice at the time of euthanasia. * $p < 0.05$.
